# Recombinant Neuregulin 1 Does Not Activate Cardiomyocyte DNA Synthesis in Normal or Infarcted Adult Mice

**DOI:** 10.1371/journal.pone.0115871

**Published:** 2014-12-29

**Authors:** Sean Reuter, Mark H. Soonpaa, Anthony B. Firulli, Audrey N. Chang, Loren J. Field

**Affiliations:** 1 The Krannert Institute of Cardiology, and the Riley Heart Research Center, Wells Center for Pediatric Research, and Indiana University School of Medicine, Indianapolis, Indiana, United States of America; 2 Department of Physiology, University of Texas Southwestern Medical Center, Dallas, Texas, United States of America; Academia Sinica, Taiwan

## Abstract

**Objectives:**

Neuregulin 1 signaling plays an important role in cardiac trabecular development, and in sustaining functional integrity in adult hearts. Treatment with neuregulin 1 enhances adult cardiomyocyte differentiation, survival and/or function *in vitro* and *in vivo*. It has also been suggested that recombinant neuregulin 1β1 (NRG1β1) induces cardiomyocyte proliferation in normal and injured adult hearts. Here we further explore the impact of neuregulin 1 signaling on adult cardiomyocyte cell cycle activity.

**Methods and Results:**

Adult mice were subjected to 9 consecutive daily injections of recombinant NRG1β1 or vehicle, and cardiomyocyte DNA synthesis was quantitated via bromodeoxyuridine (BrdU) incorporation, which was delivered using mini-osmotic pumps over the entire duration of NRG1β1 treatment. NRG1β1 treatment inhibited baseline rates of cardiomyocyte DNA synthesis in normal mice (cardiomyocyte labelling index: 0.019±0.005% vs. 0.003±0.001%, saline vs. NRG1β1, P<0.05). Acute NRG1β1 treatment did result in activation of Erk1/2 and cardiac myosin regulatory light chain (down-stream mediators of neuregulin signalling), as well as activation of DNA synthesis in non-cardiomyocytes, validating the biological activity of the recombinant protein. In other studies, mice were subjected to permanent coronary artery occlusion, and cardiomyocyte DNA synthesis was monitored via tritiated thymidine incorporation which was delivered as a single injection 7 days post-infarction. Daily NRG1β1 treatment had no impact on cardiomyocyte DNA synthesis in the infarcted myocardium (cardiomyocyte labelling index: 0.039±0.011% vs. 0.027±0.021%, saline vs. NRG1β1, P>0.05).

**Summary:**

These data indicate that NRG1β1 treatment does not increase cardiomyocyte DNA synthesis (and consequently does not increase the rate of cardiomyocyte renewal) in normal or infarcted adult mouse hearts. Thus, any improvement in cardiac structure and function observed following neuregulin treatment of injured hearts likely occurs independently of overt myocardial regeneration.

## Introduction

Many forms of cardiovascular disease are associated with acute or chronic cardiomyocyte loss. Although the adult mammalian heart retains a limited potential for regenerative growth (via proliferation of pre-existing cardiomyocytes and/or *de novo* cardiomyogenic differentiation), the magnitude of this activity has been the subject of considerable debate [1], [2]. The prevalence of myocardial insufficiency in diseased hearts underscores the reality that the intrinsic regenerative capacity of the adult heart is insufficient to repair substantive injury. Considerable effort has therefore been invested to develop interventions aimed at limiting the loss of at risk cardiomyocytes, and at enhancing the function of surviving cardiomyocytes in diseased hearts.

The neuregulins are a family of cytokines which signal through the ErbB family of tyrosine kinase receptors [3]–[6]. There are four neuregulin genes, each of which can give rise to multiple cytokines via alternative splicing. Ablation of the Neuregulin 1 gene [7], [8], the neuregulin 1 receptor ErbB4 [9], or the ErbB4 hetero-dimerizing partner ErB2 [10] resulted in aborted trabecular growth which was accompanied by embryonic lethality, suggesting that neuregulin 1 signaling might regulate cardiomyocyte proliferation during early cardiac development. Although this view was supported by several cell culture studies [11], [12], subsequent gene targeting experiments suggested that neuregulin 1 regulates cardiomyocyte differentiation and maturation during early development [13], [14].

It is also apparent that neuregulin 1 signaling plays an important role in post-natal cardiac function [15]. Although mice with cardiac-restricted ablation of the ErbB2 [16] or ErbB4 [17] receptor were normal at birth, they developed lethal dilated cardiomyopathy in adult life. Moreover, down-regulation of ErbB2/4 was observed in rats with pressure overload-induced heart failure [18]. Similarly, decreased myocardial ErbB2 and ErbB4 signaling was observed in failing human myocardium [19], and receptor levels were observed to normalize following mechanical unloading [20]. It is also noteworthy that breast cancer patients treated with Herceptin/Trastuzmab (an inhibitory ErbB2 antibody) were more susceptible to developing cardiomyopathy, particularly when co-treated with anthracycline [21], [22].

Collectively, these studies indicate that decreased neuregulin signaling is associated with adverse cardiac function in post-natal hearts. This view is supported by the observation that increasing neuregulin signaling has a positive impact on cardiomyocytes. For example, treatment with recombinant neuregulin 1 increased expression of genes associated with enhanced cardiomyocyte survival and/or function *in vitro* and *in vivo* [23]–[28]. Neuregulin treatment attenuated doxorubicin-induced cardiotoxicity [29], [30], and improved cardiac function in myocardial infarction, viral myocarditis and rapid pacing heart failure models [31]. These findings prompted several clinical trials, which to date have suggested that neuregulin treatment may improve cardiac function in patients with chronic heart failure [32], [33]. It has also been suggested that treatment with recombinant NRG1β1 (comprising neuregulin 1 amino acid residues 176–256) induced cardiomyocyte proliferation in adult mice [34] with no impact on cardiomyogenic stem cell activity, raising the possibility that enhanced cardiomyocyte renewal might underlie some of the beneficial effects of neuregulin 1 treatment in patients. In contrast, a subsequent study suggested that NRG1β1 promoted myocardial renewal *in vivo* via a combination of cardiomyogenic stem cell activation and cell cycle induction [35], although issues regarding the fidelity of the assay used to detect cardiomyocyte renewal in that study have previously been raised [36].

In this report, we further examined the impact of NRG1β1 treatment on cardiomyocyte renewal by monitoring DNA synthesis using either bromodeoxyuridine (BrdU, delivered via implanted osmotic mini-pumps) or tritiated thymidine (^3^H-Thy, delivered via IP injection) incorporation. The experiments employed transgenic mice expressing a cardiomyocyte-restricted, nuclear localized reporter to facilitate accurate cardiomyocyte nuclear identification in tissue sections. NRG1β1 treatment inhibited baseline rates of cardiomyocyte DNA synthesis in normal mice, and had no impact on cardiomyocyte DNA synthesis at the infarct border zone at 7 days post-injury. These results suggest that any benefits on cardiac structure and function observed following NRG1β1 treatment occur independently of enhanced cardiomyocyte renewal.

## Methods

### Mice

MHC-nLAC transgenic mice [37] utilize the mouse alpha-cardiac MHC promoter to target expression of a nuclear-localized β-galactosidase reporter to cardiomyocytes. Experimental mice were generated in an inbred DBA/2J background; non-transgenic breeding mates were obtained from the Jackson Laboratory (Bar Harbor, Maine). Experiments were initiated when mice reached 12 weeks of age. Experimental mice were treated with recombinant human NRG1β1 (corresponding to the EGF domain, amino acid residues 176–256, #396-HB, R&D Systems, Minneapolis, MN), at a dose of 2.5 micrograms per mouse per IP injection, dissolved in saline containing 0.1% Bovine Serum Albumin (BSA); control mice received vehicle alone. Ethics statement: all animal manipulations were performed in accordance with National Institutes of Health Guidelines and were approved by the Institutional Animal Care and Use Committee (Study #10286). All surgeries were performed under isoflurane anesthesia, and all efforts were made to minimize suffering.

### Myocardial Infarction

Myocardial infarction (MI) was performed as described previously [38]. Briefly, the animals were intubated and ventilated with 2% isoflurane and supplemental oxygen. Depth of anesthesia was monitored via tail pinch and stretch reflex. Via left thoracotomy, the left coronary artery was ligated at the inferior border of the left auricle and the animals allowed to recover for 24 hours with supplemental oxygen.

### Cardiomyocyte DNA Synthesis Assay

For BrdU labeling, mice were implanted with osmotic mini-pumps (Alzet, #1002, 0.25 microliter/hour, Palo Alto, California) containing BrdU (Roche #280879, Indianapolis, Indiana) at a concentration of 16 mg/ml in physiologic saline. Minipump implantation was as described previously [39]. Hearts were harvested after nine days of BrdU labeling, fixed in 4% paraformaledhyde, and were then embedded in paraffin and sectioned at 10 microns using standard methods [40]. Sections were subjected to antigen retrieval by incubation in sodium citrate buffer (0.01 M Tri-sodium citrate, 0.05% TWEEN 20, pH 6.0) for 30 minutes at 100°C. Non-specific immune reactivity was blocked using a M.O.M. detection kit (Vector Laboratories, Burlingame, California), and sections were then processed for β-galactosidase (Life Technologies #A-11132 rabbit anti β-galactosidase, Carlsbad, California) and BrdU (Roche #11296736001 mouse monoclonal anti BrdU) immune reactivity. Signal was developed using Alexa 555-conjugated goat anti rabbit and Alexa 488-conjugated goat anti mouse antibodies (Life Technologies, #A21429 and #A11001, respectively). Cardiomyocyte DNA synthesis was identified by the co-localization of red nuclear β-galactosidase immune reactivity and green BrdU immune reactivity.

Alternatively, mice received a single injection of tritiated thymidine (^3^H-Thy, 200 µCi i.p. at 20 Ci/mM, New England Nuclear, Boston, Massachusetts). Hearts were harvested 4 hours later, immersion fixed in 50 mM cacodylic acid/1% paraformaldehyde, cryoprotected in 30% sucrose, embedded and sectioned at 10 microns using standard histologic techniques [40]. Sections were reacted with 1 mg/ml 5-bromo-4-chloro-3-indolyl-β-D-galactoside (X-GAL) in 5 mM potassium ferricyanide, 5 mM potassium ferrocyanide, 2 mM magnesium chloride, 1x PBS. The sections were counter-stained with Hoechst 33342 (Invitrogen, Carlsbad, CA), and autoradiographic emulsion was applied and processed as described previously [41]. Cardiomyocyte DNA synthesis was identified by the co-localization of blue nuclear β-galactosidase activity and silver grains.

### Western blot analyses

Hearts were homogenized in NET buffer (150 mM NaCl, 5 mM EDTA, 50 mM Tris pH 8.0, 1% NP-40) containing protease (Roche #11 836 170 001, Indianapolis, Indiana) and phosphatase (Thermo Scientific #78420, Rockford, Illinois) inhibitors, and protein content was quantitated using the Coomassie Blue method (Pierce, Rockford, IL) as described [42]. Samples were denatured in sodium dodecyl sulfate (SDS)-polyacrylamide gel electrophoresis (PAGE) loading buffer for 5 min at 95°C and resolved on 10% SDS-PAGE gels. Fractionated proteins were then electrotransferred from the gel to nitrocellulose (Amersham) filters in Towbin buffer at 200-mA constant current and analyzed by Western blotting. The filters were stained with 0.1% naphthol blue-black in 45% methanol, 10% acetic acid to assess the efficiency of transfer. Antibodies used recognized Erk1/2 p42/p44 and p-Erk1/2[Thr^202^/Thy^204^] (#s 9102 and 4377, respectively, Cell Signaling, Danvers MA). To detect phosphorylation of cardiac myosin regulatory light chain (RLC), tissue was snap frozen in liquid nitrogen and thawed/homogenized directly in 10% tricholoro acetic acid (TCA)/10 mM DTT. Acid-precipitated proteins were washed free of TCA with ethyl ether and processed for urea/glycerol PAGE as previously described [43]. Total urea-solubilized samples (4 µg) were separated by urea/glycerol PAGE. Phosphorylated and non-phosphorylated RLC were measured by immunobloting with a total myosin antibody (Enzo, F109 3E1), used at 1∶5000 dilution in 3% BSA.

### Dispersed cell analyses

Isolated cardiomyocytes were prepared by retrograde perfusion with collagenase [39]. Animals were heparinized (10 ml/kg ip, Sigma, St. Louis, MO) approximately 5 minutes prior to sacrifice. Hearts were removed and then hung by the aorta on 23 gauge cannulae, and perfused with phosphate buffered saline (PBS) followed by 0.17% collagenase (Type I, Worthington Biochemical, Freehold NJ) in PBS. Hearts were perfused until flaccid, and ventricular cells obtained by removing the lower 75% of the heart, mincing the tissue with scissors, and then triturating with a Pasteur pipette. Cell suspensions were immediately placed in several volumes of 50 mM cacodylic acid/1% paraformaldehyde. After fixation, the cell suspensions were filtered through a fine mesh and reacted with X-GAL for 2 hours at 37°C, and then washed three times in PBS. The cell suspensions were then incubated in block solution (phosphate-buffered saline containing 0.1% Tween 20, 1% BSA and 10% goat serum) for one hour, followed by incubation with anti-cardiac alpha-actinin antibody (# A7811, Sigma-Aldrich, St. Louis MO) at a 1∶500 dilution in block solution for one hour, followed by incubation with goat anti-mouse Alexa 555 (#A-21137, Invitrogen) at a 1∶10 dilution in block solution for 1 hour. Cells were washed 3x with PBS between incubations. After processing, the cells were smeared onto positively charged slides (Superfrost Plus, Fisher, Pittsburgh, PA), and allowed to dry.

### Statistics

Results were expressed as mean ± SEM. Comparisons between two groups were performed using the unpaired Student’s t test. All tests were two-tailed. Data were considered statistically significant at p<0.05.

## Results

**Figure 1:**
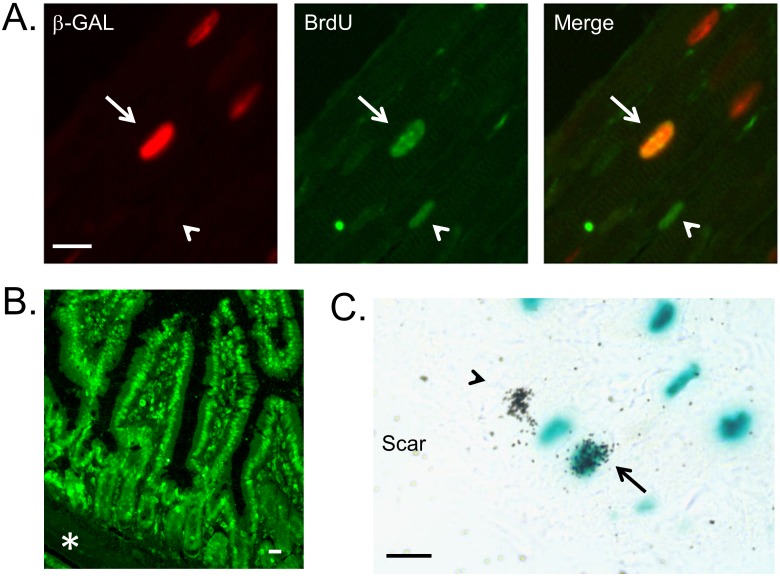
Examples of cardiomyocyte DNA synthesis assay. (A) Use of BrdU to monitor cardiomyocyte DNA synthesis in non-injured adult mice receiving 9 consecutive daily injections of NRG1β1 (BrdU was delivered using a mini-osmotic pump). Left panel shows anti-β-galactosidase immune reactivity, middle panel shows anti-BrdU immune reactivity, and right panel shows the merged image. Arrow indicates a BrdU positive cardiomyocyte nucleus, arrowhead indicates a BrdU positive non-cardiomyocyte nucleus. Bar = 10 microns. (B) BrdU incorporation in the nuclei of the small intestine microvilli epithelial cells of an NRG1β1-treated mouse. Note the absence of BrdU signal in the muscularis mucosae zone (asterisk). Bar = 10 microns. (C) Use of ^3^H-Thy to monitor cardiomyocyte DNA synthesis in non-injured adult mice receiving 9 consecutive daily injections of NRG1β1 (^3^H-Thy was delivered as a single bolus 1 hour after the last NRG1β1 treatment). Arrow indicates a ^3^H-Thy positive cardiomyocyte nucleus, arrowhead indicates a ^3^H-Thy positive non-cardiomyocyte nucleus. Bar = 10 microns.

MHC-nLAC mice, which are maintained in an inbred DBA/2J genetic background, were used to monitor the impact of NRG1β1 on cardiomyocyte DNA synthesis. When used in conjunction with BrdU incorporation, cardiomyocyte DNA synthesis is identified by the co-localization of red anti-β-galactosidase and green anti-BrdU immune reactivity in tissue sections. Adult MHC-nLAC mice were implanted with mini-osmotic pumps containing BrdU. The mice then received a total of 9 consecutive daily injections of recombinant NRG1β1 (2.5 µg/injection I.P.; control mice received vehicle only). Hearts were harvested 5 hours after the last injection, sectioned, and the sections processed for immune reactivity. Examples of DNA synthesis as detected by this assay are shown in Fig. 1A. Surprisingly, there was a reduction in the number of ventricular cardiomyocyte nuclei synthesizing DNA in mice receiving NRG1β1 as compared to mice receiving vehicle alone (Table 1, Experiment 1). To confirm BrdU delivery, small intestine from the NRG-treated mice was harvested and processed for anti-BrdU immune reactivity (the rapid turn-over of intestinal microvilli epithelium provides a convenient control for the presence of modified nucleotide [44]). BrdU signal was readily detected from the crypt to the tip of the villi (Fig. 1B).

**Table 1:** Cardiomyocyte DNA synthesis in adult MHC-nLAC mice following vehicle or NRG1β1 injection.

Experiment;Mouse Treatment;NucleotideDelivery Method	GeneticBkg.*	NRG1β1 µg/Injection	BrdU^+^or ^3^H-Thy^+^CMNuclei/Total	PositiveNuclei ±SEM (%)	# MiceAnalyzed	p vs.Control
Experiment #1;nine daily NRG1β1injections inuninjured mice;BrdU mini-pump	DBA	0	36/187,169	0.019±0.005	7	Control
	DBA	2.5	7/207,490	0.003±0.001	7	<0.05
Experiment #2;nine daily NRG1β1injections inuninjured mice; ^3^H-Thy injection onday nine	DBA	2.5	1/182,420	0.0005±0.0004	5	Control
	F1	2.5	1/216,192	0.0005±0.0006	3	>0.05
Experiment #3;three dailyNRG1β1 injectionsin uninjured mice;^3^H-Thy injectionon day three	DBA	7.5	2/383,919	0.0005±0.0002	3	>0.05†
Experiment #4;seven dailyNRG1β1 injectionsin MI mice; ^3^H­Thy injectionon day seven	DBA	0	9/23,181	0.039±0.011	5	Control
	DBA	2.5	8/29,463	0.027±0.021	6	>0.05

*Genetic background, DBA = DBA/2J; F1 = [C57Bl/6J×DBA/2J]F1.

† >0.05 vs. mice receiving 9 injections of lower dose of NRG1β1, followed by a single injection of ^3^H-Thy.

To determine if genetic background might influence the response to NRG1β1 treatment, cardiomyocyte DNA synthesis was compared in mice with DBA/2J vs. [C57Bl/6J×DBA/2J] F1 backgrounds. The mice received 9 consecutive daily injections of recombinant protein; ^3^H-Thy was injected one hour after the final NRG1β1 treatment. The hearts were harvested 4 hours later, sectioned, stained with the chromogenic β-galactosidase substrate X-GAL and processed for autoradiography. When MHC-nLAC mice are analyzed in conjunction with ^3^H-Thy incorporation and autoradiography, cardiomyocyte DNA synthesis is identified by the co-localization of the blue X-GAL reaction product and silver grains (Fig. 1C). No difference in cardiomyocyte DNA synthesis was observed in NRG1β1 treated mice with DBA/2J vs. [C57Bl/6J×DBA/2J]F1 genetic backgrounds (Table 1, Experiment 2). To determine if higher levels of NRG1β1 would promote cardiomyocyte DNA synthesis, MHC-nLAC mice (DBA/2J background) were given 3 consecutive daily injections of a 3-fold greater dose of recombinant protein. ^3^H-Thy was injected one hour after the final NRG1β1 treatment and the hearts were harvested 4 hours later and processed. No increase in ventricular cardiomyocyte DNA synthesis was detected in mice with the higher NRG1β1 dose as compared to the lower dose (Table 1, Experiment 3).

**Figure 2:**
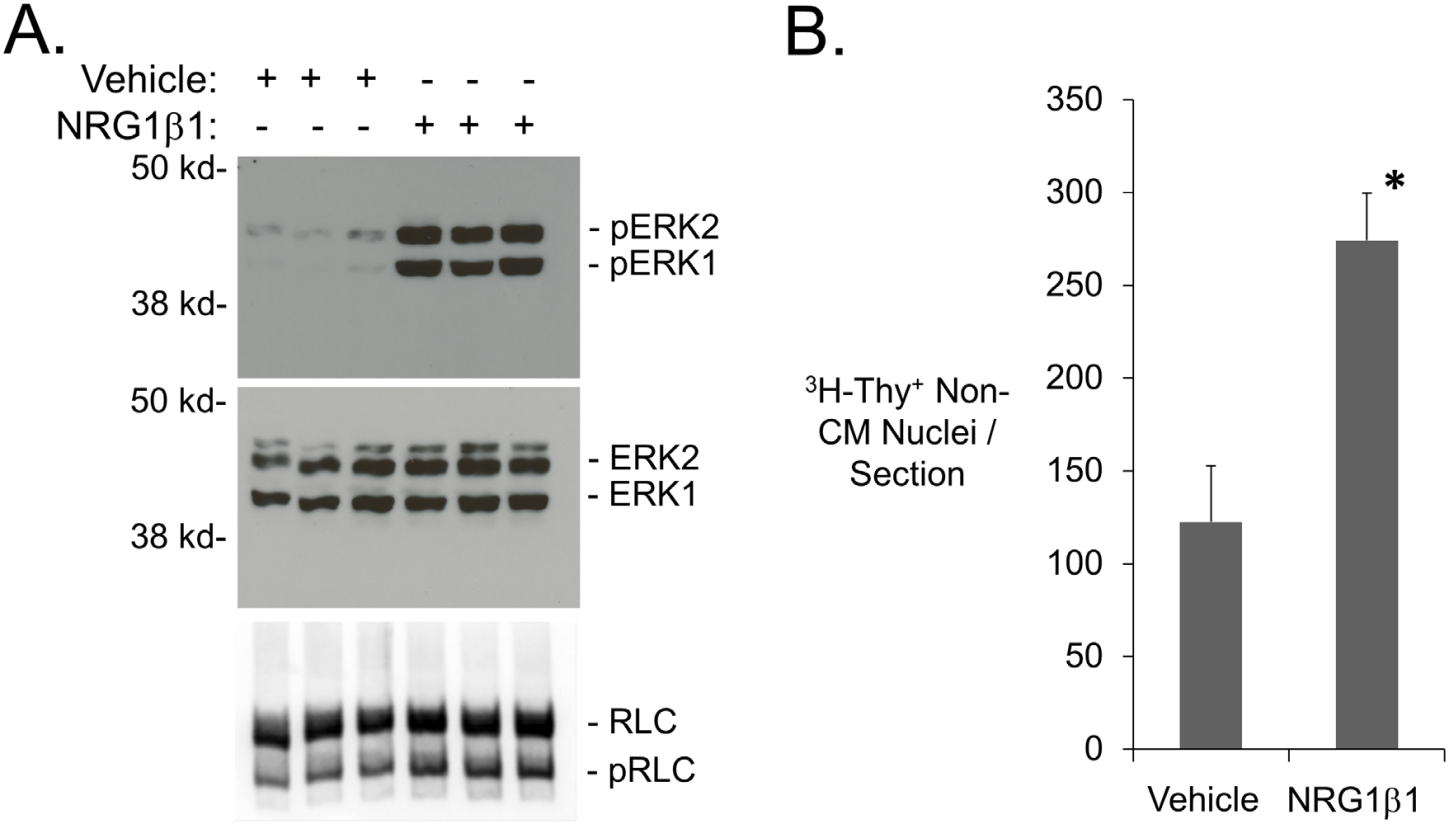
NRG1β1 elicits biological responses in the adult mouse heart. (A) Western blot demonstrating the levels of total Erk1/2 p42/p44, P-Erk1/2[Thr^202^/Thy^204^] and RLC in mice treated with NRG1β1 or vehicle (hearts harvested and processed 90 minutes after treatment). Densometric quantitation revealed that NRG1β1 treatment resulted in a 987% increase in the level of ERK1 phosphorylation, a 5727% increase in the level of ERK2 phosphorylation, and a 21% increase in the level of phosphorylated RLC vs. vehicle-treated mice (p<0.01, Student’s t-test). (B) Non-cardiomyocyte ^3^H-Thy nuclear labeling index in non-injured adult mice following 9 consecutive daily injections of NRG1β1 (5 sections analyzed from each of 4 independent mice) or vehicle (4 sections analyzed from each of 4 independent mice). *: p<0.05 vs. vehicle treated animals, Student’s t-test.

**Figure 3:**
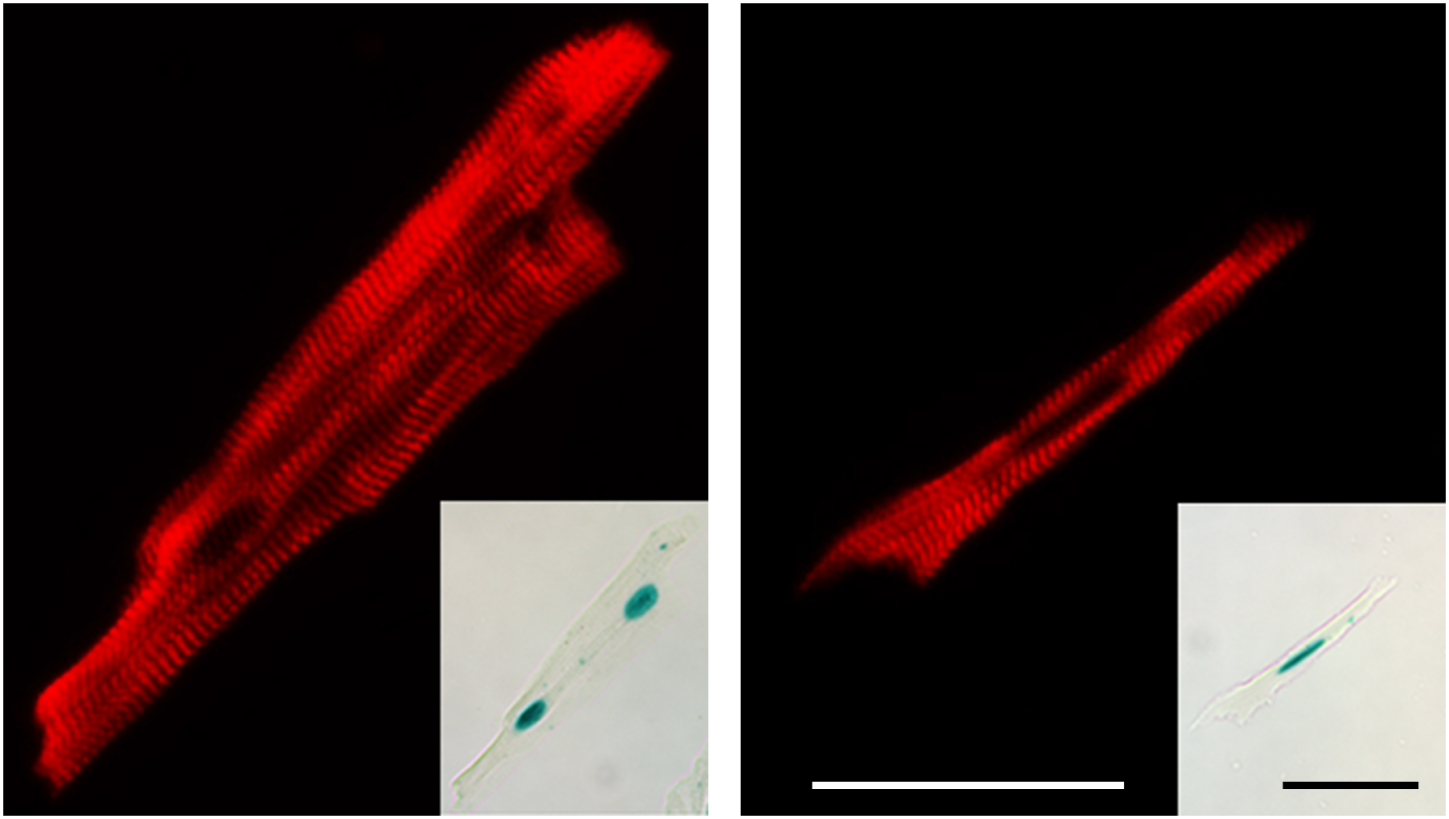
Expression of the MHC-nLAC reporter transgene following 9 consecutive daily injections of NRG1β1. Treated hearts were subjected to retrograde collagenase perfusion, and the resulting dispersed cell preparations were reacted with X-GAL and processed for cardiac alpha-actinin immune reactivity. Cardiac alpha-actinin immune reactivity (red signal) and nuclear β-galactosidase activity (inset) in a bi-nucleated (left panel) and a mono-nucleated (right panel) cardiomyocyte. Bar = 50 microns.

To confirm that NRG1β1 injection induced biological activities in our hands, mice were given a single injection of NRG1β1 (2.5 µg) and hearts were harvested 90 minutes later. Protein lysate prepared from the hearts was then processed for Western blot analyses. Previous studies utilized phosphorylation of Erk1/2 and cardiac myosin regulatory light chain (RLC) as indicators of NRG1β1 biological activity *in vivo* [31], [43]. In agreement with these previous studies, NRG1β1 treatment resulted in a statistically significant increase in the level of phosphorylation of both proteins (Fig. 2A). A 2.2-fold increase in the number of non-cardiomyocytes exhibiting DNA synthesis after 9 consecutive daily injections of NRG1β1 (analyzed by ^3^H-Thy injection at one hour after the last treatment, Fig. 2B) was observed, further indicating that NRG1β1 elicited a biological response in our hands. Previous studies demonstrated that MHC-nLAC mice have a high penetrance of transgene expression (that is, the percentage of MHC-nLAC cardiomyocytes which exhibit nuclear β-galactosidase activity). To determine if NRG1β1 suppressed the penetrance of transgene expression (which could negatively impact the ability to detect cardiomyocyte DNA synthesis in our assay), MHC-nLAC mice received 9 consecutive daily injections of recombinant protein. Five hours after the last injection, the hearts were harvested and dispersed cardiomyocyte preparations were generated via retrograde collagenase perfusion. The cells were then processed for anti-actinin immune reactivity (to identify cardiomyocytes) and X-GAL reaction (to monitor transgene penetrance). Of a total of 1,241 cardiomyocytes examined from 2 different NRG1β1-treated animals, 99.7% exhibited nuclear β-galactosidase activity (Fig. 3): 1130 of 1133 multi-nucleated (99.7%) and 107 of 108 mono-nucleated (99.1%) cardiomyocytes were X-GAL positive. These values are completely consistent with previous analyses of untreated adult MHC-nLAC mice [37]. Thus, NRG1β1 treatment did not impact the penetrance of MHC-nLAC reporter transgene expression.

To determine if NRG1β1 treatment enhances cardiomyocyte DNA synthesis in response to injury, MHC-nLAC mice were subjected to myocardial infarction (MI) via permanent coronary artery occlusion. Seven days later, consecutive daily NRG1β1 injections were initiated for a total of 7 days (control mice received injections of vehicle only). Mice received an injection of ^3^H-Thy 1 hour after the last NRG1β1 injection, and hearts were harvested 4 hours later and processed. Cardiomyocyte DNA synthesis was readily detected in the surviving LV and septum (MI border zone inclusive) of the infarcted mice, consistent with previous results [41]. However, no significant difference was observed in the percentage of ^3^H-Thy positive nuclei in mice receiving NRG1β1 as compared to mice receiving vehicle alone, although there was a trend towards a reduced cardiomyocyte labeling index in the NRG1β1-treated animals (Table 1, Experiment 4).

## Discussion

It is now well established that the normal mouse myocardium exhibits very low rates of cardiomyocyte cell cycle activity, and that this is increased following myocardial injury [41], [45]. The studies reported here demonstrate that NRG1β1 treatment inhibits the low rates of cardiomyocyte DNA synthesis present in uninjured myocardium, and furthermore fails to promote increased levels of cardiomyocyte DNA synthesis when analyzed 7 days after permanent coronary artery ligation. Since BrdU incorporated into stem cells would ultimately appear in *de novo* cardiomyoctes [46], these data also indicate that NRG1β1 does not stimulate cardiomyogenic stem cell activity over the course of the study. These data collectively suggest that NRG1β1 treatment does not promote cardiomyocyte renewal in adult mice.

This conclusion is in contrast to an earlier report suggesting that NRG1β1 induced robust cardiomyocyte cell cycle activity [34]. In that study, normal adult mice received 9 consecutive daily injections of NRG1β1 and DNA synthesis was monitored via BrdU incorporation, which was present in the drinking water during the entire treatment period. BrdU immune reactivity was reported in 14.3% of the mono-nucleated and 3% of the multi-nucleated cardiomyocytes, whereas no immune reactivity was detected in mice receiving vehicle. It was also reported that NRG1β1 treatment of mice with MI (7 consecutive daily injections initiated 7 days post-injury) resulted in a 4.4-fold increase in the level of cardiomyocyte DNA synthesis as compared to vehicle-treated animals. Although several additional experimental end points further supported the conclusion that NRG1β1 induced cardiomyocyte proliferation in the earlier study, a number of technical issues complicate critical interpretation of those data (including the potential impact of altered gene expression prior to cardiomyocyte terminal differentiation, the fidelity of the reporters used to mark cardiomyocyte nuclei in *in vitro* experiments and clonal cardiomyocyte expansion in *in vivo* experiments, a marked disconnect between the number of M-phase vs. S-phase mononuclear cardiomyocytes, and caveats regarding the age of analyses in some of the experiments [34]). Thus, the most compelling observation from the earlier study was the increased level of cardiomyocyte DNA synthesis in NRG1β1-treated, genetically naïve mice under baseline conditions and following MI. It was, however, rather surprising that the reported level of cardiomyocyte DNA synthesis in uninjured NRG1β1-treated mice was more than 10-fold greater than that in infarcted NRG1β1-treated mice (when normalized for the difference in the duration of BrdU treatment). Indeed, myocardial injury is typically associated with an increase in cardiomyocyte cell cycle activity [41], [45].

Given these discrepant results, it is important to critically examine the technical aspects of the current study. The observation that cardiomyocyte DNA synthesis was reproducibly detected in uninjured, vehicle-treated hearts argues that both the BrdU and the ^3^H-Thy assays were sufficiently sensitive to detect any NRG1β1-induced increase in cell cycle activity. Indeed, the failure to detect BrdU incorporation in vehicle treated uninjured hearts in the earlier study [34] suggests that the DNA synthesis assays employed in the current study were more sensitive. The observation that a three-fold increase in NRG1β1 concentration failed to induce cardiomyocyte DNA synthesis suggests that the animals were not simply under-dosed. The observation that similar results were obtained with [C57Bl/6J×DBA/2J]F1 animals suggests that genetic background was not a major contributor to the absence of DNA synthesis. The high penetrance of reporter transgene expression (only ca. 0.1% of the mono-nucleated and 0.3% of the multi-nucleated cardiomyocytes lacked β-galactosidase activity) argues that, if NRG1β1 treatment of uninjured mice induced cardiomyocyte DNA synthesis as was reported previously (i.e., in 14.3% of the mono-nucleated and 3% of the multi-nucleated cardiomyocytes [34]), the vast majority of these cells would have to also express the MHC-nLAC reporter and thus would have been detectable by our assay system.

It is of interest to note that the BrdU experiments used mice maintained in a DBA/2J genetic background; 7.8% of the cardiomyocytes are mono-nucleated in this background [39]. We screened 207,490 cardiomyocyte nuclei in tissue sections from un-injured mice treated with NRG1β1; we would anticipate approximately 16,184 of these nuclei were from mono-nucleated cells, and that 191,306 were from bi- or multi-nucleated cardiomyocytes. Only 7 BrdU positive cardiomyocyte nuclei were detected of the 207,490 nuclei screened. Even if all 7 BrdU cardiomyocyte nuclei were present in the mono-nucleated pool, this would account for only 0.043% of the population (7 nuclei of 16,184 total mono-nuclear cardiomyocyte nuclei), which is 333-fold lower than the rate of 14.3% reported by Kuhn and colleagues. Similarly, if all 7 BrdU cardiomyocyte nuclei were present in the bi/mulinucleated-nucleated pool, this would account for only 0.0037% of the population (7 nuclei of 191,306 total bi/multi-nuclear cardiomyocyte nuclei), which is 810-fold lower than the rate of 3% reported by Kuhn and colleagues. Thus, the frequency of cardiomyocyte DNA synthesis in NRG1β1-treated animals in our study is more than two orders of magnitude lower that that reported by Khun’s group, irrespective of the affected sub-population (i.e., mono- vs. bi/multi-nucleated).

It is important to note that delivery of NRG1β1 to uninjured hearts did in fact elicit a number of biological responses. Increases in the level of phosphorylated Erk1/2 and cardiac myosin regulatory light chain were observed, in agreement with previous studies examining the impact of treatment on uninjured hearts. Moreover, NRG1β1 treatment increased the levels of non-cardiomyocyte DNA synthesis. These observations suggest that the recombinant protein was biologically active in our hands when delivered to uninjured mouse hearts. A limitation of the current study is that the long-term impact of NRG1β1 treatment on post-MI cardiac function was not monitored. However, this shortcoming does not impact on the results obtained studying uninjured hearts.

In light of these observations, it is difficult to identify technical deficiencies to explain the absence of NRG1β1-induced cardiomyocyte DNA synthesis in the current study. It should however be noted that the MHC-nLAC mice have been used extensively to track cardiomyocyte DNA synthesis during post-natal development, following intra-cardiac transplantation, following myocardial injury and in genetically modified animals [37], [41], [42], [47]–[52]. Given this, the simplest interpretation of the current results is that that NRG1β1 treatment does not result in an increase in the number of cardiomyocytes exhibiting DNA synthesis, and consequently does not increase the rate of cardiomyocyte renewal, in normal or injured adult mouse hearts. Ultimately, the ability to accurately quantitate the impact of NRG1β1 (or any other intervention) on cardiomyocyte cell cycle activity, and consequently the impact on myocardial renewal, is dependent upon the rigor of the assays employed to identify cardiomyocytes (or cardiomyocyte nuclei) and to document cell cycle activity. In light of the arguments raised above regarding the fidelity of the MHC-nLAC reporter, the data presented here, albeit negative, are compelling and difficult to discount.

## Conclusions

As indicated above, a substantial body of preclinical data, as well as preliminary clinical trials in humans, suggests that NRG1β1 treatment can have a positive impact on cardiac function following myocardial injury. Understanding the underlying molecular mechanism for short- and long-term NRG1β1-mediated functional improvement could provide important insight for improving treatment efficacy. Given the data presented here, any beneficial impact of NRG1β1 treatment in injured hearts is not attributable to enhanced myocardial regeneration.
